# Optimizing lumbar therapy: a biomechanical study of oblique pulling manipulation under different postures

**DOI:** 10.1186/s12906-026-05477-1

**Published:** 2026-07-27

**Authors:** Yanfei Wang, Aorui Yu, Lianhai Jin, Weidong Liu, Dayu Chen, Yafei Cao, Kun Gao

**Affiliations:** 1 The Fourth Clinical Medical College of Guangzhou University of Chinese Medicine, Guangzhou, Shenzhen 518033 China; 2 Shenzhen Pingle Orthopaedic Hospital Affiliated to Guangzhou University of Traditional Chinese Medicine, Shenzhen, China; 3 Department of Joints and Soft Tissue Injury, Shenzhen Traditional Chinese Medicine Hospital, Shenzhen, 518033 China; 4 Department of Orthopedics, Shenzhen Traditional Chinese Medicine Hospital, Shenzhen, 518033 China

**Keywords:** Lumbar disc herniation, Oblique pulling manipulation, Infrared optical motion capture, Finite element modeling, Biomechanical experiments

## Abstract

**Background:**

While oblique pulling manipulation has proven to be an effective conservative treatment approach for lumbar disc herniation, it is not without limitations. One of the primary drawbacks of this technique is the potential for inaccurate localization during the procedure. This study aims to explore the effects of the oblique pulling manipulation under different lumbar postures and to characterize the resulting load distribution across different lumbar regions.

**Methods/study design:**

This study employed a multi-modal approach combining infrared optical motion capture, finite element modeling, and biomechanical experiments. Eighteen healthy volunteers were divided into three groups for motion capture analysis of the oblique pulling manipulation at different angles. A finite element model of the lumbar spine was developed based on CT scans of a 24-year-old male volunteer and validated against existing literature. Biomechanical experiments were conducted on six cadaveric specimens using a BOSE testing machine to simulate the manipulation technique. Pressure measurements in the intervertebral discs and facet joints were obtained using a Tekscan pressure sensor.

**Results:**

The experimental results showed that the biomechanical effects of oblique pulling manipulation were closely related to lumbar posture. Infrared optical motion capture, finite element modeling, and biomechanical experiments consistently demonstrated posture-dependent, region-specific loading patterns, with peak forces preferentially concentrated around the L3/4 region in extension, the L4/5 region in flexion, and the L5/S1 region in excessive flexion. These findings suggest that adjusting lumbar posture may influence the regional distribution of mechanical loads and provide a biomechanical basis for posture-guided application of oblique pulling manipulation.

**Conclusion:**

These findings provide a biomechanical basis for posture-guided application of oblique pulling manipulation and may assist clinicians in selecting appropriate manipulation postures for patients with lumbar disc herniation.

## Introduction

Lumbar disc herniation (LDH) is a common clinical degenerative spinal disorder that significantly affects patients’ quality of life and work capacity [1, 2]. In recent years, the incidence of LDH has been increasing, especially among younger populations. According to epidemiological studies, the global prevalence of LDH is approximately 1%-3%, with a slightly higher rate of 4.8% in China [3, 4]. Chinese chiropractors in Hong Kong have successfully employed spinal manipulation and mobilization, for several decades in treating LDH [5, 6]. Traditional Chinese bone-setting techniques, particularly the lumbar spine oblique pulling manipulation - a method sharing similarities with certain chiropractic adjusting techniques - have gained prominence due to their significant immediate effects, ease of operation, and cost-effectiveness [7, 8]. Recent systematic reviews have shown that bone-setting techniques are more effective than conventional conservative treatments in relieving pain and improving function in LDH patients [7, 9, 10].

However, traditional lumbar spine oblique pulling manipulation still has some limitations, primarily in terms of force distribution and inaccurate reduction positioning, which make it difficult to preferentially concentrate mechanical loads around the affected lumbar region. Han et al. [11], Jin [12]. Optimizing the distribution of mechanical loads during oblique pulling manipulation has become an important research focus. Clinically, variations in hip flexion-extension and lower limb positioning appear to influence the direction and distribution of mechanical forces transmitted to the lumbar spine. Previous biomechanical studies have demonstrated that changes in lower limb posture can alter lumbar segmental motion, facet joint loading, and intervertebral stress distribution, thereby potentially enabling preferential mechanical loading of affected lumbar regions in patients with lumbar disc herniation (LDH).Mechanistically, this posture-dependent regional loading pattern can be explained by the “Paper Strip Theory” proposed by Kang [13]: when a curled paper strip is twisted at both ends, a new curvature replaces the original one and is necessarily coupled with rotation. Applied to the lumbar spine, altering hip flexion and lower-limb positioning changes the spinal curvature, and the resulting bending–rotation coupling caninfluence the regional distribution of mechanical forces within the lumbar spine.This provides a qualitative basis for the notion that precise adjustment of lumbar posture during oblique pulling manipulation can achieve region-specific mechanical effect [13]. Based on these biomechanical principles and our clinical observations, controlled adjustment of hip motion and lower limb angle during manipulation may contribute to preferential regional load concentration and local pain relief in LDH patients [14–16].

Although clinical observation suggests that hip-flexion angle during oblique pulling may influence the regional distribution of mechanical loads within the lumbar spine, this concept lacks systematic biomechanical validation [8]. We hypothesized that controlled changes in lumbar posture would produce differential stress distributions across L3/4, L4/5, and L5/S1 regions. The present multi-modal study combined infrared optical motion capture, finite element modeling, and cadaveric pressure measurements to test this hypothesis and provide an evidence-based framework for posture-guided load distribution in lumbar disc herniation.Infrared motion capture was selected for its ability to track real-time, high-precision displacement of lumbar segments during manipulation, overcoming the spatial and temporal limitations of traditional video analysis. Finite element modeling was chosen to determine stress distributions and displacements across lumbar segments under varying postures. In vitro cadaveric experiments were performed to directly measure intradiscal and facet joint pressures, bridging the gap between computational predictions and physiological reality. The aim of this study was to determine whether specific lumbar positioning produces distinct region-specific loading patterns for the management of lumbar disc herniation (LDH), and to investigate the associated biomechanical effects on on different lumbar regions. This research will not only help optimize the clinical application of the oblique pulling manipulation but also provide a solid scientific foundation for this traditional treatment approach, supporting a more rational and evidence-based clinical application in the treatment of LDH and, given the widespread use of lumbar manipulation for low back pain of various etiologies, informing the management of other lumbar pathologies as well.

## Materials and methods

### Infrared optical motion capture system

#### Experimental subjects

Eighteen young volunteers (9 males and 9 females) were recruited from the Fourth Clinical Medical College of Guangzhou University of Chinese Medicine (Shenzhen Traditional Chinese Medicine Hospital) to participate in this study. A sample size of 18 volunteers was determined to provide 80% power to detect a medium effect size (Cohen’s d = 0.5) with a significance level of 0.05. Volunteers were randomly assigned to one of three groups (extension, flexion, excessive flexion) using a computer-generated randomization sequence. The randomization was stratified by gender to ensure equal distribution of males and females across groups. All participants voluntarily signed informed consent forms after being fully informed about the study.

The following exclusion criteria were applied: Participants with a history of lumbar spine injury, discomfort, or conditions leading to limited lumbar spine mobility; Positive results in the bent-back test or straight leg raise test, or suspected spinal injury with spinal cord damage; Lumbar instability or developmental spinal canal stenosis; Participants with severe heart, lung, brain, or hematologic diseases, or those suffering from tumors, tuberculosis, osteomyelitis, or osteoporosis.

#### Measurement equipment and process

Field calibration was performed by starting the motion capture system (RTS2000, REALIS, China), and sequentially use the L-shaped and T-shaped calibrators for static and dynamic calibration within the field to calibrate the instrument and field. Marker Point Setup: Set specific marker points on the L3/4 intervertebral space, L4/5intervertebral space, L5/S1 intervertebral space. Place specially designed illuminated markers on the exposed skin over the spinous processes as marker points. Motion capture was performed by activating the optical motion capture system and simulate the rotational lifting technique. The RTS2000 system featuring 2 million pixels and a maximum capture frame rate of 340 frames per second, forms an optical motion capture system to establish a high-precision motion capture environment. This process is dynamically recorded by the optical motion capture system, and data is analyzed and processed using the RTS Rovonga software.

The RTS2000 infrared optical motion capture system was validated prior to data collection. Calibration was performed using L-shaped and T-shaped calibrators to ensure spatial accuracy within 0.1 mm. The system’s accuracy was further confirmed by comparing the measured displacements of known distances with their actual values, resulting in a mean error of less than 0.05 mm.

#### The oblique pulling manipulation

**Fig. 1:**
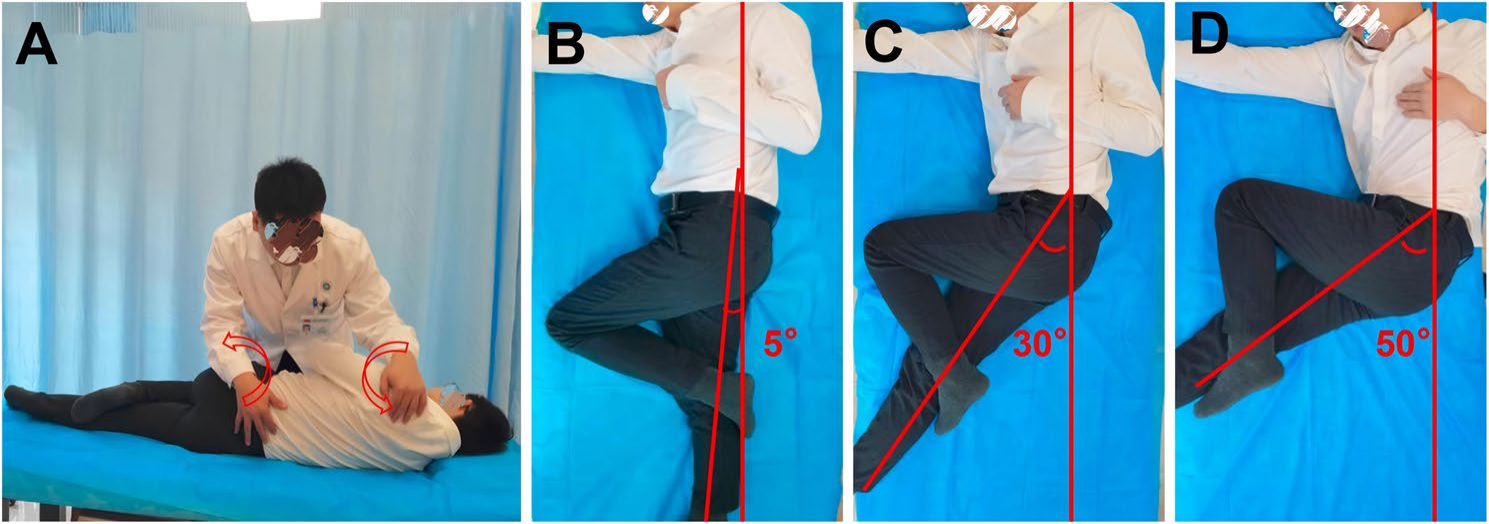
Procedure of the oblique pulling manipulation and different body postures. **A** The operation of the oblique pulling manipulation. **B **Patient posture in the extension position. **C **Patient posture in the flexion position. **D **Patient posture in the excessive flexion position

The procedure was performed by a senior chiropractor specializing in spinal manipulation. Initially, the volunteers were positioned prone, and muscle relaxation techniques were applied to release tension in the lumbar and back muscles. Subsequently, the oblique pulling manipulation was applied to the lumbar spine. During the treatment, the volunteer was placed in a lateral recumbent position. The upper leg was flexed at the hip and knee to 90°, with the ankle placed in the popliteal fossa of the lower leg. The lower leg was adjusted to different angles depending on the group: in the extension position, the angle was 5°; in the flexion position, it was 30°; and in the excessive flexion position, it was 50° (Fig. 1). The chiropractor placed one elbow on the anterior side of the patient’s shoulder, while the other hand supported the pelvis. Both elbows applied equal force but in opposite directions, performing a push-pull maneuver. When resistance was felt, the push-pull force was suddenly increased, often accompanied by a “clicking” sound, though this was not forced.

### Finite element model

#### Experimental subject

A 24-year-old male volunteer was recruited from the Fourth Clinical Medical College of Guangzhou University of Chinese Medicine (Shenzhen Traditional Chinese Medicine Hospital) to participate in the study. The volunteer’s height was 180 cm, and mass was 75 kg. X-ray examination ruled out spinal infections, tumors, and scoliosis. He had no history of spinal surgery or trauma.

#### Geometric modeling

CT scanning was performed in the supine position along the longitudinal axis of the body using a 64-slice spiral CT scanner (Siemens, Germany). The scanning range covered the lumbar spine, pelvis, and hip joints, with a slice thickness set to 1.5 mm. The CT data were imported into Mimics software (Mimics 21.0, Materialise, Belgium) to generate an initial 3D mesh model. Using grayscale value recognition, masks were created to separate bone from soft tissue. The model was then refined by removing noise, filling cavities, and isolating the lumbar spine, pelvis, and hip joints. The final 3D model was exported as an STL file and further optimized in Geomagic software (Geomagic 2021, Geomagic, USA) to ensure smooth surfaces and accurate anatomical representation.

#### Meshing strategy

The optimized model was imported into SolidWorks software (SolidWorks 2022, Dassault Systems, United States) for assembly and error surface checks. The 3D model was imported into Ansys software (Ansys 23.0, Ansys, USA) for meshing. A tetrahedral mesh was applied to the lumbar spine, pelvis, and hip joints, with an average element size of 1.5 mm. Mesh quality was assessed using metrics such as aspect ratio, skewness, and Jacobian, ensuring that all elements met the criteria for accurate finite element analysis. The final mesh consisted of approximately 500,000 elements and 1,200,000 nodes.A mesh convergence study was conducted by gradually refining the element size from 2.0 mm to 1.0 mm. Convergence was confirmed when further refinement below 1 mm resulted in less than 3% change in peak von Mises stress; the final mesh (approximately 500,000 elements, 1,200,000 nodes) was therefore considered adequate for analysis.

DICOM images were imported into Mimics and oriented to generate a three-dimensional view. Thresholding based on Hounsfield Unit (HU) values was applied to separate bone from surrounding soft tissues. A threshold range of 220–1800 HU was used to reconstruct the overall vertebral bony structure. For soft tissue, a range of − 100 to + 200 HU was applied to capture intervertebral discs, ligaments, and surrounding musculature. The threshold values were subsequently verified against the gray-value histogram of the CT data and adjusted via profile line analysis to ensure accurate tissue boundary demarcation. The resulting bone mask underwent noise removal, bone segmentation, isolation of individual bone blocks, cavity filling, and removal of unwanted regions. After the finalized mask was solidified into a preliminary three-dimensional model, cortical and cancellous bone were differentiated using a geometry-based approach: a uniform cortical shell with a thickness of 1 mm was generated by applying an inward offset from the external vertebral surface, while the remaining internal volume was defined as cancellous bone [17]. The resulting cortical and cancellous geometries were then exported as STL files. These STL files were imported into Geomagic software, where mesh optimization—including noise reduction, refinement, and feature surface fitting—was performed to meet the requirements of subsequent finite element analysis, and the optimized model was saved as an STP file.

#### Material property selection

Material properties were assigned to the model, and mesh quality was adjusted to ensure the accuracy of the analysis. The following thicknesses were set: endplate thickness of 0.1 mm, cortical bone thickness of 0.4 mm, and the nucleus pulposus occupying 41% of the intervertebral disc volume. The lumbar ligament and pelvic ligament were modeled as 3D truss components using tension only, with attachment points determined based on anatomical positions and cross-sectional areas defined according to the literature. The facet joint surfaces were set as nonlinear 3D contact, using surface-to-surface contact elements. The superior and inferior articular processes, as well as the cartilage, were defined as frictional contacts with a friction coefficient of 0.1 [18–20]. The material properties assigned to the model are listed in Table 1. Finally, loading conditions were defined within the Ansys work environment, and static structural analysis was completed, with relevant post-processing results extracted. Boundary conditions were applied to simulate the three postures: extension, flexion, and excessive flexion. The inferior surface of the S1 vertebra was fixed, and a rotational moment of 10 Nm was applied to the L1 vertebra to simulate extension. The hip joints were modeled as fixed to simulate the patient’s lower limb position during the oblique pulling manipulation.

**Table 1:** Material properties assigned to different anatomical regions

Material Name	Young’s Modulus (MPa)	Poisson’s Ratio
Pelvic Cortical Bone	17,000	0.3 [21]
Pelvic Cancellous Bone	10	0.2 [22]
Cartilage	25	0.4 [23]
Nucleus Pulposus	2	0.499 [22]
Annulus Fibrosus	8.4	0.45 [24]
Endplate	500	0.25 [23]
Vertebral Cortical Bone	12,000	0.3 [22]
Vertebral Cancellous Bone	100	0.2 [22]

### Finite element model validation

The generated INP file was imported into Abaqus software for calculation and analysis. A vertical downward concentrated load of 500 N was first applied, along with a 10 Nm torque in different directions to simulate the loading conditions of the lumbar spine under flexion, extension, lateral bending, and rotation. Next, the stress distribution in the intervertebral discs and pelvis under these loads was closely examined.

To validate the accuracy of the finite element model, the simulated range of motion and peak von Mises stress were compared with published cadaveric data [25–27]. And a mesh convergence study confirmed that refining element size to < 1 mm resulted in < 3% change in peak stress, making it suitable for subsequent biomechanical simulations and analysis.

During the validation phase, particular attention was given to the model’s range of motion, stress distribution, and the responses of the intervertebral discs and pelvis under different loading conditions. Through this detailed comparison, the goal was to ensure that the model not only correctly reflects the experimental data but also accurately simulates the biomechanical properties of the lumbar spine in practical applications. If the validation results show high reliability and accuracy, the model can then be used for more complex simulation scenarios and research.

### Mechanical characteristics of the oblique pulling manipulation

We analyzed the mechanical characteristics of the oblique pulling manipulation using a finite element model. Loading was set according to the clinical procedure steps, with the model positioned at three angles: flexion, extension, and excessive flexion. Based on the method outlined by Zhang et al., [27, 28], the following mechanical loads were applied to the model: a maximum impact force of 190 N was applied at the iliac crest, while a 190 N remote force was applied at the top of the L1 vertebra, in the opposite direction of the impact force.We analysed the overall stress in the lumbar spine and pelvis, as well as the stress on the intervertebral discs and facet joint cartilage, under the effect of the oblique pulling manipulation at different angles.

### Biomechanical experiment

#### Preparation of experimental specimens

Six fresh cadaveric specimens from the lumbosacral region were obtained from the Department of Human Anatomy, Guangzhou University of Traditional Chinese Medicine. The specimens were dissected to remove skin, subcutaneous tissue, and paraspinal muscles while preserving the ligamentous structures of the intervertebral joints. The T12-S2 segments were excised and wrapped in two layers of preservation film to maintain moisture. Specimens were stored at -20 °C and thawed overnight at 4 °C prior to testing. During the experiment, the specimens were intermittently sprayed with phosphate-buffered saline (PBS, pH 7.4) to maintain hydration.

One day prior to the experiment, the specimens were removed from the freezer and placed in the refrigeration compartment to thaw naturally overnight. After thawing, the T12 vertebra at the upper end and the S2 vertebra at the lower end of each specimen were placed into square molds and embedded with polymethyl methacrylate (PMMA, New Century Dental Materials Co., Ltd., Shanghai, China), leaving the L1-S1 segment exposed.

#### Lumbar spine manipulation simulation

The experiment was conducted at room temperature (approximately 25 °C). The prepared lumbar spine specimens were fixed to a BOSE dynamic/static material testing machine (BOSE ELECTROFORCE Model 3510-AT, BOSE, USA) with a square clamp at the head end and the tail end fixed to a vice. To minimize the viscoelastic effects on the specimen, a small-range preload/unload treatment was applied prior to the experiment. A 10 N•m torque was applied for 10 s, followed by unloading, and the rotational angle was recorded. A 3-minute interval was allowed between each loading cycle to allow the specimen to return to its physiological state [29]. The loading/unloading cycle of 10 N•m torque was repeated until three consecutive cycles resulted in the same rotational angle (± 0.1°). The BOSE testing machine used angle control mode with the following parameters: Preload angles: 0°, 10°, and 20°, simulating patient postures of extension, flexion, and excessive flexion, respectively. Preload rotation angle: 15°, speed: 3°/s. Maximum load angle: 20°, speed: 33°/s. Return speed: 3°/s. Horizontal loading of 22 N was applied to the L5 spinous process. A downward load of 300 N was applied to simulate the upper body mass.

#### Pressure measurement

A fine needle was used to sequentially puncture the anulus fibrosus of the lumbar intervertebral disc and the joint capsule of the zygapophysial joints, following the measurement sequence. A Tekscan 6900 and a Tekscan I-Scan 5076 pressure sensor (Tekscan, USA), each wrapped in plastic film, were inserted respectively into the right lumbar facet and the joint space and the intervertebral disc anulus fibrosus. The other end of the sensor was connected to the Tekscan handle. The Tekscan pressure distribution measurement system was activated to collect stress data from the intervertebral disc and facet joint during the manipulation simulation.

### Statistical analysis

Statistical analyses were conducted using SPSS 21.0 (IBM Corp., USA). Normality of the data was assessed via the Shapiro-Wilk test (α = 0.05), and homogeneity of variances was confirmed using Levene’s test. For normally distributed data, results are presented as mean±standard deviation (SD).In the human experiment, segmental displacement, intradiscal pressure, and facet joint pressure were compared among the three lumbar postures (extension, flexion, and excessive flexion) using one-way analysis of variance (ANOVA). Post-hoc pairwise comparisons were conducted with the least significant difference (LSD) test, and the Bonferroni correction was applied to adjust for multiple comparisons.In the finite element analysis, stress distributions and displacements at the target and adjacent lumbar segments were compared across the same postural conditions using analogous parametric or non-parametric approaches according to the data characteristics.For the cadaveric validation, the intradiscal and facet joint pressures measured experimentally were directly compared with the corresponding finite element predictions to quantify model accuracy.Non-normally distributed data were analyzed using the Kruskal-Wallis test, with Mann-Whitney U tests for pairwise comparisons and Bonferroni-adjusted significance levels. A *p*-value of less than 0.05 was considered statistically significant.

## Result

### Intervertebral displacement across lumbar segments in three postures

**Fig. 2:**
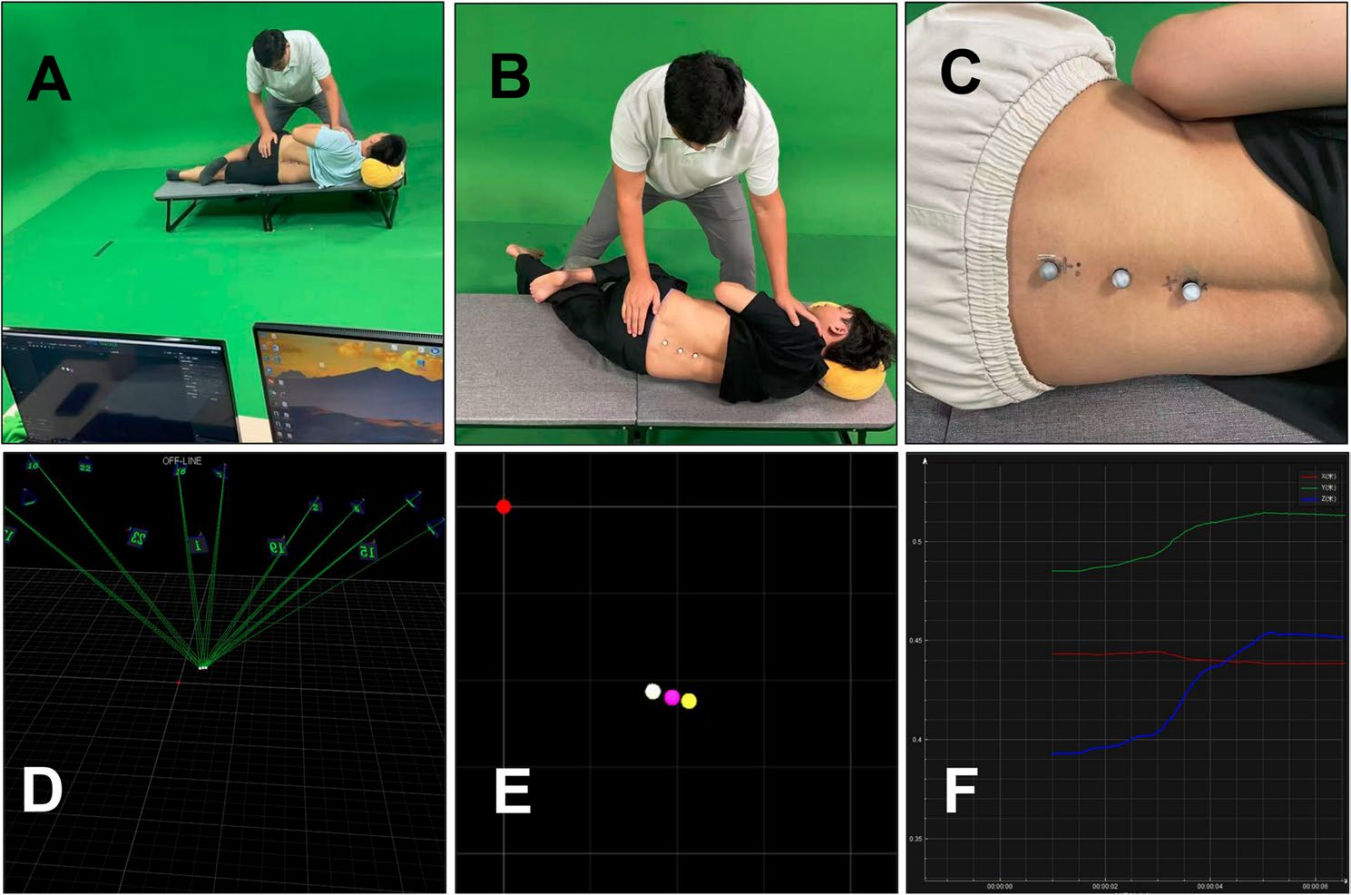
Displacement of lumbar vertebrae measured by the motion capture system under the force of oblique pulling manipulation. **A**-**C** Demonstration of the manipulation and the labeling of marker points. **D**-**F **Displacement curves of the marker points

The displacement of the intervertebra l spaces was measured using a motion capture system under oblique pulling manipulation (Fig. 2). Smaller displacement indicates more concentrated force application.In the extension position, displacement increased progressively from L3/4 to L5/S1 (L3/4: 18.44 ± 2.45 mm; L4/5: 29.76 ± 1.71 mm ; L5/S1: 42.50 ± 2.06 mm; all *P* < 0.05). In the flexion position, the L4/5 intervertebral space showed the smallest displacement ༈15.66 ± 3.16 mm ༉, which was significantly less than that of L3/4 and L5/S1 (*P* < 0.05). In the excessive flexion position, the smallest displacement shifted to L5/S1 ༈18.81 ± 2.84 mm ༉, significantly less than that of L3/4 and L4/5 (*P* < 0.05) (Table 2) .

**Table 2:** Displacement of intervertebral spaces at different lumbar segments under the force of oblique pulling manipulation

Position	Extension (mm) (*N* = 6)	95%CI	Flexion (mm) (*N* = 6)	95%CI	Excessive Flexion (mm) (*N* = 6)	95%CI
L3/4 Intervertebral Space	18.44 ± 2.45	[16.32, 20.56]	34.62 ± 4.18	[30.34, 38.90]	47.18 ± 2.63	[44.39, 49.97]
L4/5 Intervertebral Space	29.76 ± 1.71①	[28.18, 31.34]	15.66 ± 3.16①	[12.33, 18.99]	41.12 ± 2.85	[38.12, 44.12]
L5/S1 Intervertebral Space	42.50 ± 2.06①②	[40.21, 44.79]	35.72 ± 3.63②	[31.98, 39.46]	18.81 ± 2.84①②	[15.81, 21.81]
F-value	216.700		59.712		224.600	
*P*-value	< 0.001		< 0.001		< 0.001	

①Significantly different from the L3/4 intervertebral space within the same posture (*P* < 0.05)

②Significantly different from the L4/5 intervertebral space within the same posture (*P* < 0.05)

### Finite element analysis of peak stress distribution

**Fig. 3:**
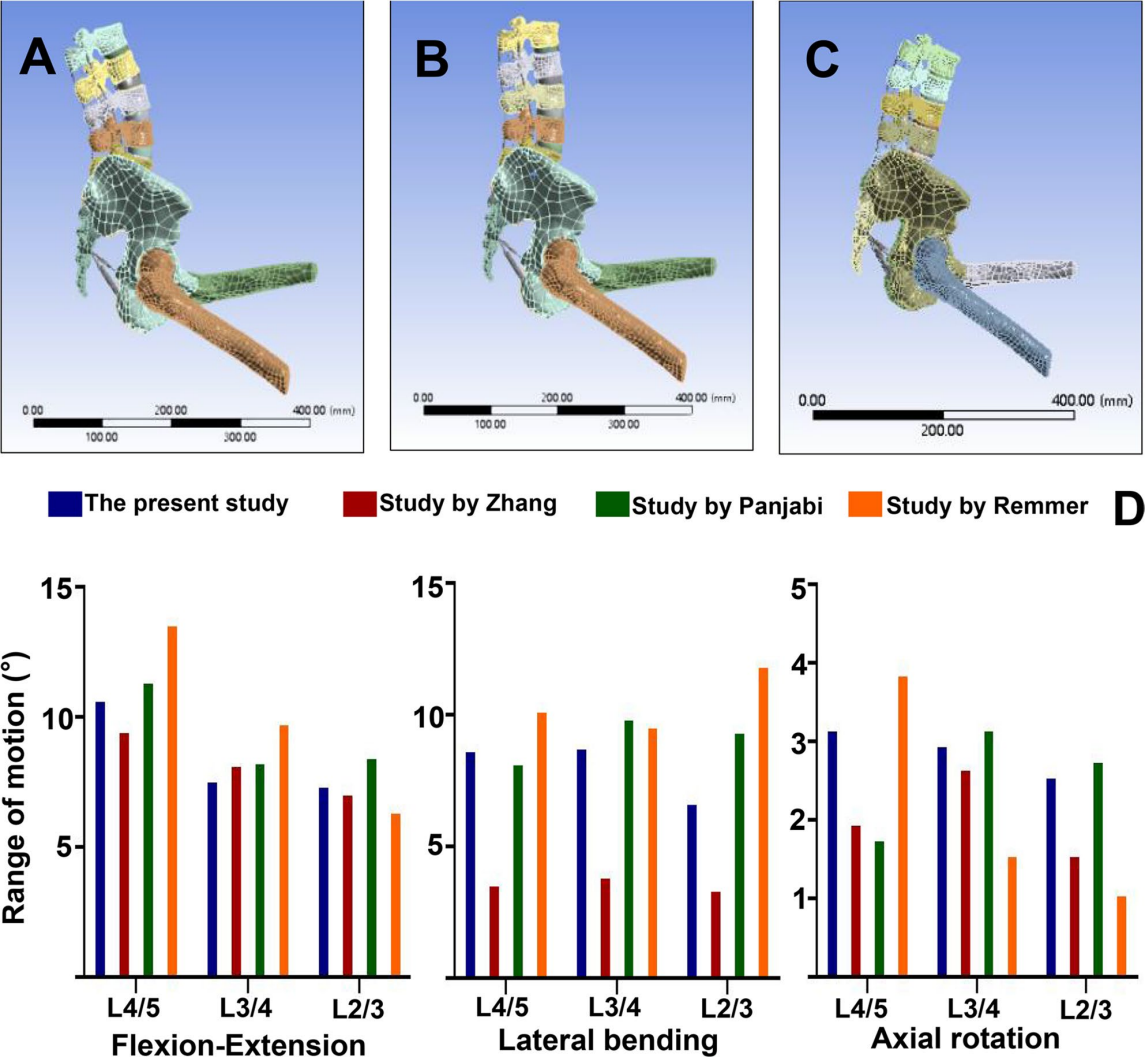
Construction and validation of the 3D finite element model of the Lumbar-Pelvis-Hip system. **A**-**C **Models in extension, flexion, and excessive flexion positions, respectively. **D **Comparison of the range of motion (°) at three lumbar segments

To investigate the peak stress distribution under different lumbar postures, A three-dimensional finite element model of the lumbar spine, pelvis, and hip joint was successfully constructed. The model includes key structures such as the lumbar vertebrae, pelvis, hip joints, intervertebral discs, and articular cartilage. The range of motion (ROM) of the model was analyzed under different loading conditions, including flexion, extension, and axial rotation, and compared with experimental data from relevant literature [25, 30]. The results suggest there is a similar trend in the model’s behavior from L2/L3 to L4/L5, which aligns with the findings in the literature.This validates the reliability and applicability of the model, making it suitable for further biomechanical analysis (Fig. 3).

Following validation of the three-dimensional finite element model against published data, the peak stresses on the intervertebral disc and facet joints were analyzed (Table 3). In extension, both disc and facet joint stresses decreased segmentally from L3/4 to L5/S1 (disc: 4.45, 3.87, and 2.87 MPa; facet joint: 2.91, 2.09, and 1.49 MPa). In flexion, peak stresses concentrated at the L4/5 segment (disc: 4.79 MPa; facet joint: 3.24 MPa). In excessive flexion, the peak stresses shifted to the L5/S1 segment (disc: 5.05 MPa; facet joint: 4.11 MPa).

**Table 3:** Peak stress in intervertebral discs and facet joints at different positions in the lumbar-pelvis-hip 3D finite element model

Segment	Lumbar Disc Pressure Peak (MPa)			Facet Joint Pressure Peak (MPa)		
	Extension	Flexion	Excessive Flexion	Extension	Flexion	Excessive Flexion
L3/4	4.45	3.37	3.29	2.91	2.52	2.69
L4/5	3.87	4.79	4.37	2.09	3.24	3.29
L5/S1	2.87	3.51	5.05	1.49	2.27	4.11

### Biomechanical pressure measurements in cadaveric specimens

**Fig. 4:**
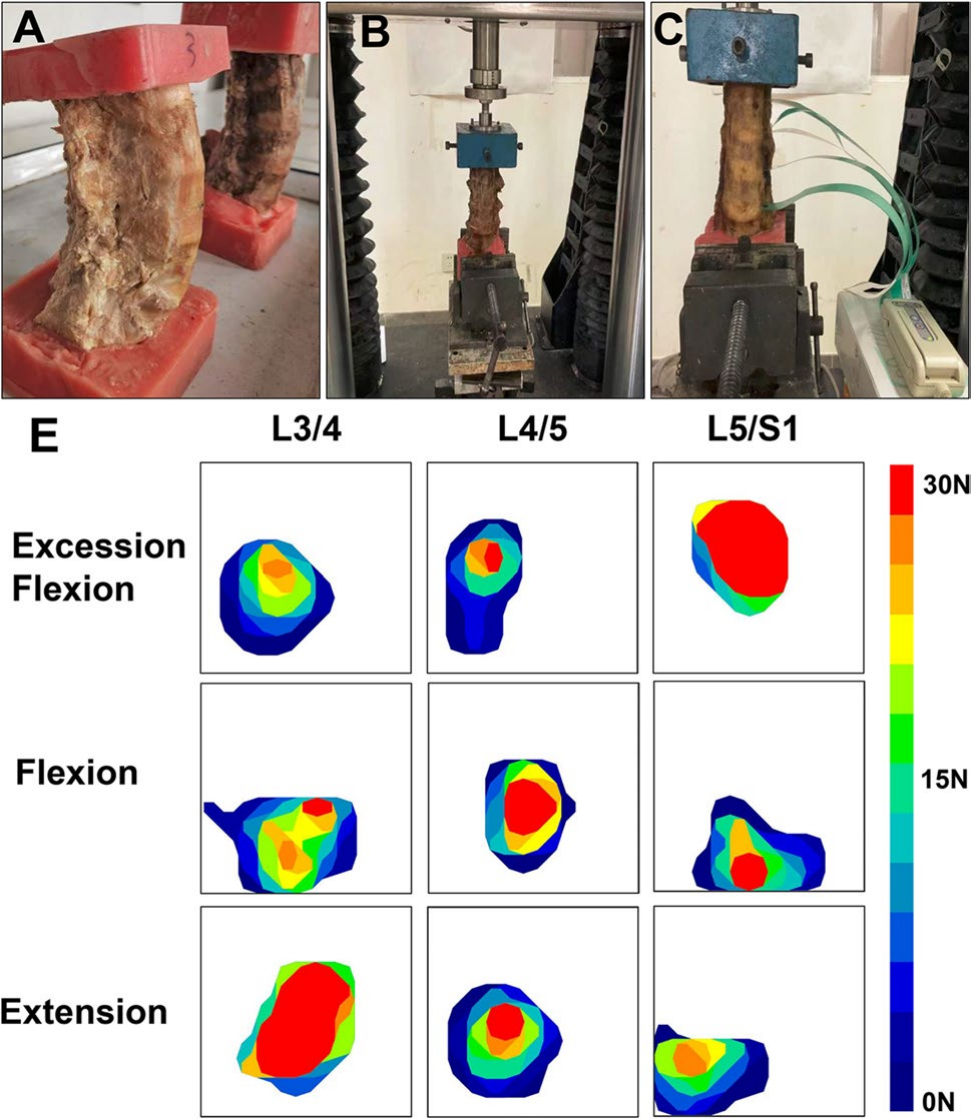
Biomechanical analysis of lumbar spine cadaveric specimens in three postures. **A**-**C **Lumbar spine specimens fixed in a BOSE dynamic/static materials testing machine, with Tekscan pressure sensors inserted into the intervertebral discs or facet joints. **D**, **E **Display of intervertebral disc and facet joints pressure values at different segments in three postures

The biomechanical pressure measurements from the cadaveric specimens were consistent with the stress distribution trends observed in the finite element analysis (Fig. 4). In the extension position, the L3/4 segment sustained the highest average pressure (disc: 37.49 ± 4.58 N ; facet joint: 17.23 ± 1.10 N ), followed by L4/5, with L5/S1 showing the lowest values (all *P* < 0.05). In the flexion position, the highest pressure was recorded at the L4/5 segment (disc: 39.61 ± 3.69 N; facet joint: 18.51 ± 1.49 N ), which was significantly greater than that at L3/4 and L5/S1 (*P* < 0.05). In the excessive flexion position, the L5/S1 segment bore the greatest pressure (disc: 40.95 ± 3.93 N; facet joint: 19.65 ± 1.74 N), while L3/4 pressure was significantly higher than L4/5 (disc: 26.45 ± 2.70 N vs. 25.65 ± 2.40 N; facet joint: 11.07 ± 1.71 N vs. 10.90 ± 1.83 N; both *P* < 0.05) (Table 4).

**Table 4:** The average pressure in the intervertebral disc and right-sided facet joint cadaveric specimens

Segment	Average Pressure Value of the Lumbar Intervertebral Disc (*N*)						Average Pressure Value of the Facet Joint (*N*)					
	Extension (*N* = 6)	95%CI	Flexion (*N* = 6)	95%CI	Excessive Flexion (*N* = 6)	95%CI	Extension (*N* = 6)	95%CI	Flexion (*N* = 6)	95%CI	Excessive Flexion (*N* = 6)	95%CI
*L3/4*	37.49 ± 4.58	[32.33, 42.65]	27.60 ± 2.93	[24.61, 30.61]	26.45 ± 2.70	[23.61, 29.29]	17.23 ± 1.10	[16.11, 18.35]	12.88 ± 1.51	[11.32, 14.44]	11.07 ± 1.71	[9.27, 12.89]
L4/5	25.72 ± 4.08①	[21.44, 30.00]	39.61 ± 3.69①	[35.75, 43.47]	25.65 ± 2.40①	[23.13, 28.17]	13.30 ± 1.29①	[11.91, 14.71]	18.51 ± 1.49①	[16.89, 20.15]	10.90 ± 1.83①	[8.97, 12.83]
L5/S1	24.45 ± 3.64①②	[20.77, 28.13]	26.29 ± 3.48②	[22.75, 29.85]	40.95 ± 3.93①②	[36.96, 44.94]	10.60 ± 1.39①②	[9.15, 12.05]	12.44 ± 1.40②	[10.99, 13.89]	19.65 ± 1.74①②	[17.76, 21.54]
F-value	20.403		32.240		28.88		45.32		34.845		54.670	
*P*-value	<0.001		<0.001		0.000		0.000		<0.001		0.000	

①*P* VS L3/4,②*P* VS L4/5

## Discussion

This study demonstrates that oblique pulling manipulation produces posture-dependent, region-specific biomechanical effects: peak mechanical loading preferentially concentrated around the L3/4 region, L4/5 in flexion, and L5/S1 in excessive flexion. The convergent results from optical motion capture, finite element analysis, and cadaveric experiments provide a biomechanical rationale for optimizing the clinical application of this technique.

This mechanical behavior aligns with the “Paper Strip Theory” proposed by the Chinese scholar Kang Fuxiao [13]. During oblique pulling manipulation, the spine functions as a multi-segmented lever system: when torsional force is applied, the region exhibiting the smallest displacement becomes the instantaneous axis of rotation and bears the greatest mechanical load. Using an infrared optical motion capture system [31–33], we confirmed that the site of minimal displacement shifts systematically with posture—L3/4 in extension, L4/5 in flexion, and L5/S1 in excessive flexion—a pattern consistent with the theoretical prediction that spinal curvature influences the focal distribution of mechanical loads [34, 35]. The underlying mechanism involves adjusting the flexion angle of the lumbar-pelvic-hip complex: changing the rotation and flexion of the spine concentrates peak force at the curvature apex, thereby producing region-specific loading patterns. Clinically, the practitioner can modify the angle between the patient’s lower limb and the body axis in the lateral recumbent position, inducing anterior hip rotation and altering the lumbar-pelvic-hip angle. As this angle decreases, the curvature of the lumbar-pelvic-hip complex flattens and the force focus shifts cephalad; conversely, as the angle increases, the curvature increases and the force focus migrates caudally.

Although the motion capture system only permits indirect measurement of body posture, the data support our hypothesis that the focal point of force application matches our predictions. To further verify the force distribution trends, we employed a finite element model to analyze loads acting on the spine.

A three-dimensional finite element model of the lumbar spine–pelvis complex was constructed to simulate the oblique pulling manipulation in different postures. Stresses on the intervertebral discs and facet joints at various levels were quantified, and the peak stress locations were identified. The results showed that the highest peak disc stress occurred at L3/4 in extension, L4/5 in flexion, and L5/S1 in excessive flexion, with the facet joint stresses exhibiting a parallel migration pattern. The facet joints, together with the intervertebral disc, form the lumbar three-joint complex, which transmits load and maintains spinal stability by limiting the range of motion, bearing approximately 33% of the dynamic compressive load and 35% of the static load [36]. Oblique pulling manipulation may alleviate symptoms such as low back pain caused by lumbar disc herniation by promoting facet joint sliding and correcting zygapophyseal joint dysfunction [37]. This posture-dependent shift of peak stress corroborates the findings of Wen et al., [38], who reported significant increases in L4–L5 and L5–S1 disc stress in seated postures, with the greatest increase at L5–S1. Moreover, the finite element predictions are consistent with the cadaveric experimental results.

Previous studies have questioned the ability of spinal manipulation to produce truly segment-specific effects because externally applied forces are distributed across multiple adjacent vertebral levels [39–41]. Although our finite element results demonstrated preferential stress concentration in different lumbar regions under different manipulation postures, this does not imply exclusive loading of a single motion segment. Instead, the findings support the existence of posture-dependent, region-specific loading patterns. Future studies with larger sample sizes and in vivo validation are required to further determine the biomechanical and clinical significance of these observations.

These findings carry important clinical implications. By adjusting lumbar posture during oblique pulling manipulation, clinicians may preferentially concentrate mechanical loads within a target lumbar region, potentially improving treatment precision and efficacy. For instance, positioning a patient with an L4/5 disc herniation in flexion may increase loading around the L4/5 region and enhance the therapeutic effect. However, these data were obtained from healthy volunteers and non-degenerated cadaveric specimens; direct translation to patient populations requires prospective clinical validation.

This study also has several limitations. First, the sample size is modest (18 volunteers, 6 cadaveric specimens), which may limit the generalizability of the conclusions. Second, neither the healthy volunteers nor the cadaveric tissues underwent disc degeneration grading; their biomechanical responses may differ substantially from those of patients with lumbar disc herniation. Third, and most critically, active core musculature was omitted in both the finite element model and the ex vivo experiments. Core muscles significantly modulate spinal stiffness and load distribution during dynamic manipulation Shan et al., [42], and their absence may lead to overestimation of the precision or magnitude of force transmission. Fourth, the lack of long-term follow-up precludes assessment of sustained therapeutic effects. Future research should validate these biomechanical findings in larger patient cohorts with graded disc pathology, incorporate electromyography or dynamic muscle models to account for neuromuscular contributions, and evaluate clinical outcomes over extended follow-up periods.

## Conclusion

In conclusion, lumbar oblique pulling manipulation produces posture-dependent, region-specific biomechanical loading patterns, with peak stresses preferentially concentrated around the L3/4 region in extension, the L4/5 region in flexion, and the L5/S1 region in excessive flexion.The consistent findings obtained from motion capture, finite element analysis, and cadaveric experiments provide biomechanical evidence that lumbar posture influences the regional distribution of mechanical loads during manipulation. These findings may assist clinicians in selecting manipulation postures according to the anatomical level of interest and contribute to a more rational application of lumbar oblique pulling manipulation. Given that lumbar manipulation is used for a variety of lumbar disorders beyond lumbar disc herniation, the posture-dependent biomechanical principles identified in this study may have broader clinical relevance. Further clinical studies are required to validate these findings in patient populations.

## Data Availability

The datasets used and/or analyzed during the current study are available from the corresponding author on reasonable request.
